# Epidemiological and Clinical Characteristics of Influenza Outbreaks Among Children in Chongqing, China

**DOI:** 10.3389/fpubh.2022.760746

**Published:** 2022-04-15

**Authors:** Xuchen Meng, Han Zhao, Rong Ou, Qing Zeng, Huiqun Lv, Hua Zhu, Mengliang Ye

**Affiliations:** ^1^Department of Epidemiology and Health Statistics, School of Public Health and Management, Chongqing Medical University, Chongqing, China; ^2^Clinical College, Chongqing Medical University, Chongqing, China; ^3^Chongqing Municipal Center for Disease Control and Prevention, Chongqing, China; ^4^The Library, Chongqing Medical University, Chongqing, China; ^5^Department of Epidemiology and Health Statistics, School of Public Health and Management, Chongqing Medical University, Chongqing, China

**Keywords:** influenza, epidemiological characteristics, preschool and school-age children, influenza outbreak, spatial autocorrelation analysis (SAA)

## Abstract

Influenza is a global serious public health threat. Seasonal influenza among children in Chongqing has been a heavy health burden. To date, few studies have examined the spatial and temporal characteristics of influenza. This research sheds new light on correlating them with influenza outbreaks with data of over 5 years (2014–2018). All cluster outbreaks among preschool and school-age children reported in Chongqing were collected through the Public Health Emergency Management Information System. The demographical, epidemiological, and clinical data of the cases were analyzed. From 2014 to 2018, a total of 111 preschool- and school-based influenza-like illness outbreaks involving 3,549 cases were identified. Several clinical symptoms that were analyzed in this study showed significant contrast between influenza A and B. Spatial autocorrelation analysis over the 5-year data detected Xiushan district being the most likely cluster. The exploration of the spatial distribution and clinical characteristics of influenza cluster of children in Chongqing could help the effective implementation of health policies. Future studies should be conducted to monitor the outbreaks of influenza among children.

## Introduction

Influenza is a globally prevalent disease. It is a leading cause of annual epidemics, especially in tropical and subtropical areas. Influenza viruses that can infect humans are divided into three types (A–C), while seasonal epidemics are generally caused by types A and B. Influenza A viruses have several subtypes on the account of antigenic shift, while influenza B viruses currently have only two genetically distinct lineages (B/Yamagata and B/Victoria) (1). Though generally considered as a mild disease, according to the latest estimate, 290,000–650,000 deaths due to influenza occur each year (2). Recent studies tend to focus on differences in symptoms between influenza strains (3, 4). Still, several epidemiological characteristics between influenza A and B infections have been proven to be distinguishable (5). The present study aimed to explore further differences in clinical manifestations between the strains. Many studies have demonstrated that preschool and school-aged children are among the groups most susceptible to influenza infection. Few research directly investigated the spatial distribution of school-based cluster outbreaks (6–10). Kindergartens and primary schools are full of lively and vulnerable children. Thus, they are destined to be places where influenza outbreaks are likely to occur. Therefore, specific research is meaningful and necessary.

Chongqing is the largest subtropical municipality located in Southeast China. Influenza poses a significant public health threat to Chongqing due to the humid subtropical monsoon climate and dense population. An annual average of 10,025 influenza-associated deaths occurred in Chongqing, corresponding to 5.2% of all deaths (11). Few literature could be found on the epidemiological and virological characteristics of influenza from 2011–2015 in Chongqing (11–13).

Vaccination is considered the best preventive measure against influenza (14–16), and vaccination among school-aged children can indirectly protect other age groups (17, 18). However, it does not provide the best protection when a poor match exists between the vaccine strain and the circulating strain or when a new pandemic virus emerges (19–21). Furthermore, the duration and predominant virus types are unpredictable, making influenza vaccination extremely difficult. No previous study has employed spatial and temporal techniques for exploring the distribution and clinical features of influenza. The present paper aimed to critically analyze the epidemiological and clinical characteristics of influenza A and B among preschool and school-aged children in Chongqing from 2014 to 2018.

## Methods

### Study Setting and Population

Influenza is marked by sudden onset of fever, myalgia, headache, malaise, dry cough, sore throat, and nasal congestion (22–24). Influenza-like illness (ILI) is defined as fever (body temperature ≥ 38°C) accompanied by cough or sore throat. The onset time of fever should be within the course of this acute fever, and temperature determination includes body temperature measured by the patient and the medical institution (25, 26). Laboratory-confirmed cases are defined as those confirmed with influenza virus detected by laboratory RT-PCR from swab samples (27).

An influenza-like case outbreak is defined as an unusually high number of influenza-like cases occurring within a short period of time in the same area or unit (28). In the present study, the monitored outbreaks were defined as more than 10 cases of ILI detected in the same school, childcare institution, or other group units in 1 week (25).

**Figure 1:**
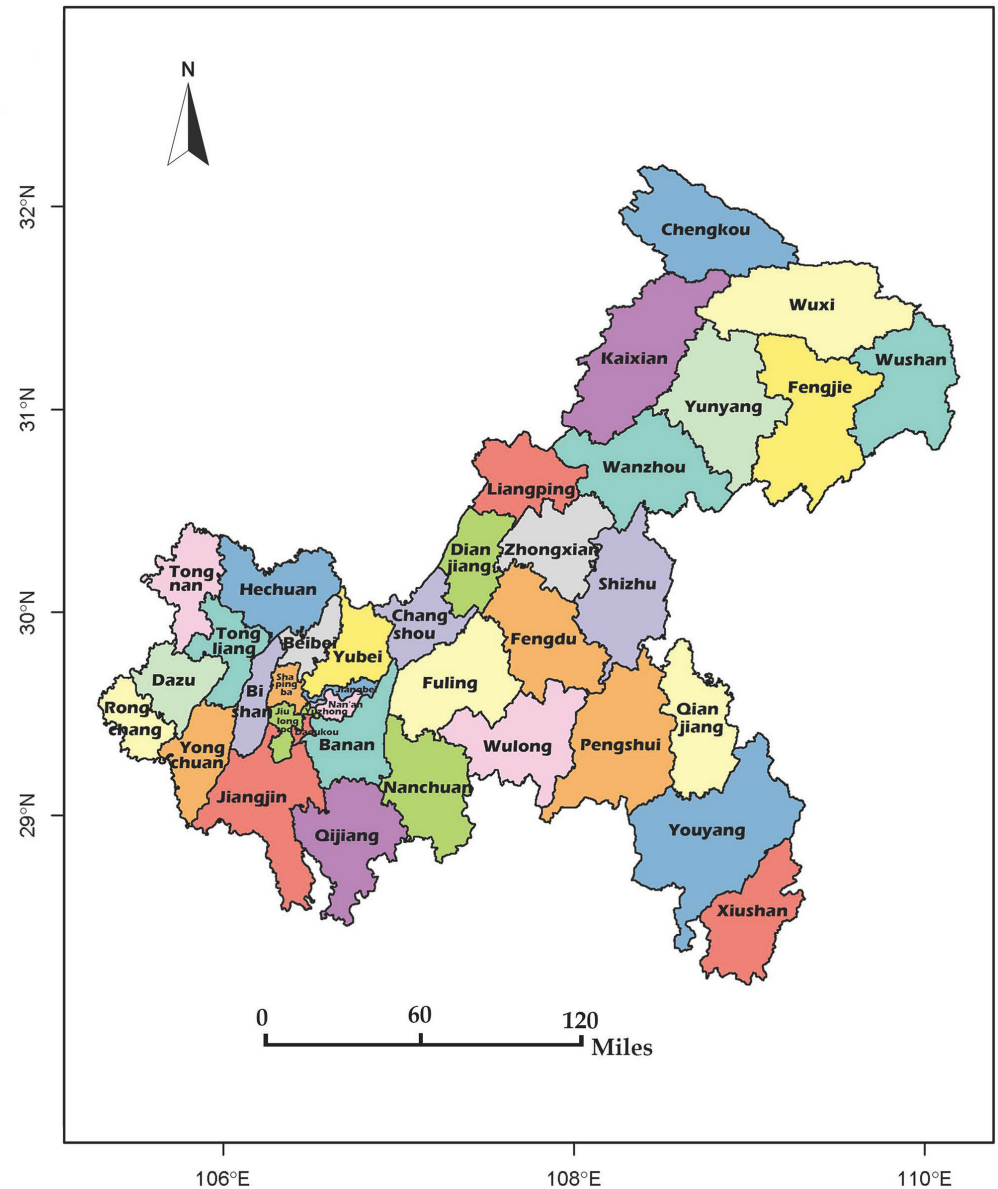
Map showing the geographical location of Chongqing and the districts in this city.

The study was conducted in Chongqing. The city location and district distributions are shown in Figure 1. Chongqing is in Southwest China and can be located at longitude 106°3448.31” E and the latitude 29°3340.37” N coordinates. Chongqing is the largest municipality of China, covering an area of 82,403 km^2^. It has 26 districts, eight counties, and four autonomous counties (Figure 1), with a permanent resident population of 31,243,200. In this study, all the districts, counties, and autonomous counties were included. This study also focused more on differences between rural and urban schools than different political systems of management. Besides, the medical systems of the districts, counties, and autonomous counties were quite similar. Therefore, all of these areas were called districts to keep the units the same.

### Data Acquisition

The Guidelines for Handling Outbreaks of Influenza-like Cases (2012 edition) were developed by the Chinese National Ministry of Health to standardize the handling and management of influenza-like cases (25). The guidelines require that if an abnormally high number of influenza-like cases occurs in an area or unit for a short period of time, they must be reported to the local disease control and prevention center. All laboratory-confirmed and clinically diagnosed cases should be reported directly through the Public Health Emergency Management Information System of the Chinese Disease Prevention and Control Information System. The data of cluster outbreaks in kindergartens and schools from January 2014 to December 2018 in Chongqing from the case network were analyzed.

For outbreaks of influenza-like cases that meet the reporting criteria, local agencies were required to collect samples of outbreak cases. Pharyngeal swabs, nasal swabs, and nasopharyngeal swabs of influenza-like cases within 3 days of onset were collected and placed into a sampling tube containing 3–4 mL sampling solution. The freshly collected specimens were transported to the influenza surveillance network laboratory within 48 h. After the samples of the outbreak were received, the influenza surveillance network laboratories were required to identify the influenza virus subtypes or lineages by means of nucleic acid test in 24 h. Real-time RT-PCR test was conducted for qualitative identification of influenza viruses in respiratory tract samples and virus-isolated cultures by using One-Step RT-PCR kit in accordance with the standardized protocols formulated by the National Technical Guidelines for Influenza Surveillance (25). The present study included all the cluster outbreaks in childcare facilities and schools in Chongqing, China, from January 2014 to December 2018.

### Statistical Analysis

The number distribution of clustered cases is described by types and years to show the trend of influenza outbreaks in Chongqing. The demographic characteristics of patients with ILI were described, and the incidence was calculated by dividing the number of cases by the number of exposures. The number of exposures is defined as the number of a school or classroom depending on the specific condition of different schools. Pearson Chi-square test was conducted to test differences in independent association between categorical variables. Fisher's exact test was employed when sample sizes within cells were small (<5). The odds ratio (OR) and 95% confidence interval (CI) was also determined using the Pearson Chi-square test and used to evaluate the likelihood of contracting influenza in the regions.

The spatial distribution of laboratory-confirmed influenza cases was shown by computing the number of cases at the district level and differently colored in the map by using the software ArcGIS10.2 (ESRI, Redlands, CA, USA). This study also utilized spatial autocorrelation analysis, with one district as a geographic unit. Global and local Moran's I were applied to identify the significant spatial autocorrelation regions of influenza in Chongqing (29). The global indicators of spatial association expresses the strength of spatial autocorrelation present in the influenza incidence across a whole aerial dataset (29). The significance of global Moran's I was validated by Monte Carlo tests with Z-scores (|Z| < 1.96) and *p*-values (*P* < 0.05). The value of Moran's I is generally between −1 and 1. I > 0 indicates positive autocorrelation of the distribution, I = 0 indicates that the spatial distribution is random, and I < 0 means that the distribution has a global negative autocorrelation. The local indicators of spatial association (LISA) is composed of spatial autocorrelation present in the influenza incidence across an aerial dataset to each of the component areas. LISA map was used to cluster the significant spatial regions and display them in significant and clustering graphs (30). When the incidence rates were similarly high or low, they were defined as having a positive autocorrelation (represented as high-high or low-low autocorrelation). If the attributes held opposing high and low values, they were considered to have a negative autocorrelation (represented as high-low or low-high autocorrelation) (31, 32).

## Results

### Epidemiological Characteristics

From January 2014 to December 2018, a total of 111 preschool- and school-based ILI outbreaks were reported in Chongqing, China. Table 1 presents an overview of the ILI cluster outbreaks over the 5 years. The clusters of influenza outbreaks were similar in 2014 and 2015, with more outbreaks of influenza A than B. The number of influenza B outbreaks exceeded that of influenza A in 2016 and 2017 and reached a 5-year peak in 2017. In 2018, the number of clusters and districts involved were the lowest.

**Table 1:** Cluster outbreaks of influenza-like illness cases.

**Year**	**Number of outbreaks (*N* = 106)**	**Type A (N1 = 47)**	**Type B (N2 = 59)**	**Negative (*N*0 = 5)**	**Number of districts involved (*n* = 38)**
2014	21	16	5	1	12
2015	21	13	8	1	13
2016	16	5	11	2	12
2017	38	5	33	1	10
2018	10	8	2	0	7

*N: total number of outbreaks among preschool and school-aged children; n: total number of Chongqing districts*.

**Figure 2:**
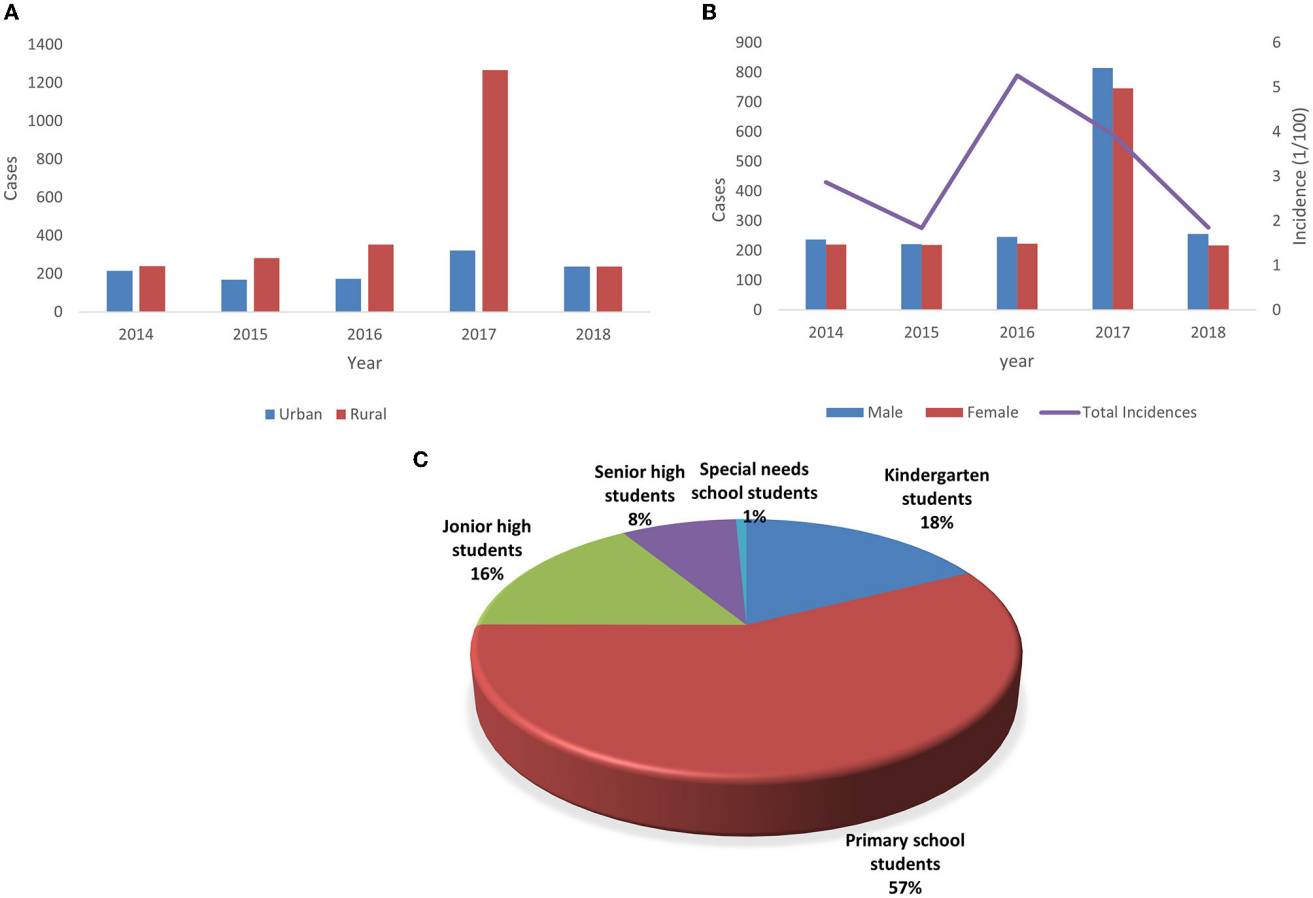
Demographic characteristics of influenza cases in Chongqing from 2014 to 2018. **(A)** Annual amount of influenza cases by gender and the annual incidence of total influenza cases. **(B)** Annual amount of influenza cases by region. **(C)** Distribution of influenza cases by educational stage.

A total of 3,549 cases of ILI were reported among preschool and school-aged children during the study period, with 3,403 laboratory confirmed cases, 145 virus-negative cases, and one fatal case. The general characteristics of all cases, including gender, incidence, region, and educational stage, are shown in Figure 2.

A total of 1,846 male ILI cases included 1,776 laboratory-confirmed cases and 70 virus-negative cases, and 1,702 female ILI cases comprised 1,627 laboratory confirmed cases and 75 virus negative cases. The ratio of male to female was 1.09:1. Boys infected with influenza A (52.8%) and B (51.7%) were slightly more than girls, while the only death in 5 years was a female student in rural primary school.

In terms of educational stage, students in special-needs school (15.82%) and those in kindergarten (9.57%) were the most affected groups. Regarding the subtypes and lineages of influenza virus, the incidence of B/Victoria (4.30%) and B/Yamagata (3.29%) was higher than that of other groups. Though 40.8% of children became infected in winter, the incidence rate of influenza in this season was the lowest (2.21%). The incidence rates of influenza A and influenza B were the highest in autumn (5.43%) and summer (5.81%), respectively. The incidence of influenza infection among children in rural areas (4.05%) was twice that in urban areas (1.90%).

Living in rural areas (OR = 2.18, 95% CI = 2.03–2.35, *P* < 0.0001) was determined to be a risk factor of influenza infection compared with living in urban areas.

### Clinical Presentation

The children infected with influenza B could easily have headache (*P* < 0.001) and dizziness (*P* = 0.002). Significant differences were found between the accompanying respiratory symptoms of influenza A and B, with cough (*P* < 0.001), nasal discharge (*P* < 0.001), and sore throat (*P* < 0.001) being more common in influenza B. Fisher's exact test showed that abdominal pain (*P* = 0.002) was significantly more frequent in patients with influenza B (Table 2).

**Table 2:** Comparison of symptoms between influenza A and B cases.

	**Type A N1 = 1,512**	**Type B N2 = 1,891**	**Total *N* = 3,403**	***P* value (Chi square)**	**Negative *N*0 = 145**
Systemic features					
headache	130 (8.60)	322 (17.03)	452 (13.28)	<0.001	11 (78.57)
myalgia	34 (2.25)	57 (3.01)	91 (2.67)	0.169	
Respiratory symptoms					
cough	246 (16.27)	936 (49.50)	1,182 (34.73)	<0.001	20 (13.79)
Sore throat	160 (10.58)	653 (34.53)	813 (23.89)	<0.001	15 (10.34)
Nasal discharge	142 (9.39)	282 (14.91)	424 (12.46)	<0.001	8 (5.52)
Nervous symptoms					
dizziness	67 (4.43)	130 (6.87)	197 (5.79)	0.002	-
weakness	45 (2.98)	49 (2.59)	94 (2.76)	0.496	-
Gastrointestinal symptoms					
diarrhea	-	5 (0.26)	5 (0.15)	0.070^Δ^	-
nausea	-	3 (0.16)	3 (0.09)	0.259^Δ^	-
vomiting	18 (1.19)	13 (0.69)	31 (0.91)	0.125^Δ^	-
Abdominal pain	-	11 (0.58)	11 (0.32)	0.002^Δ^	-

Δ *Fisher's Exact Test*.

### Temporal Distribution of Influenza A and B

**Figure 3:**
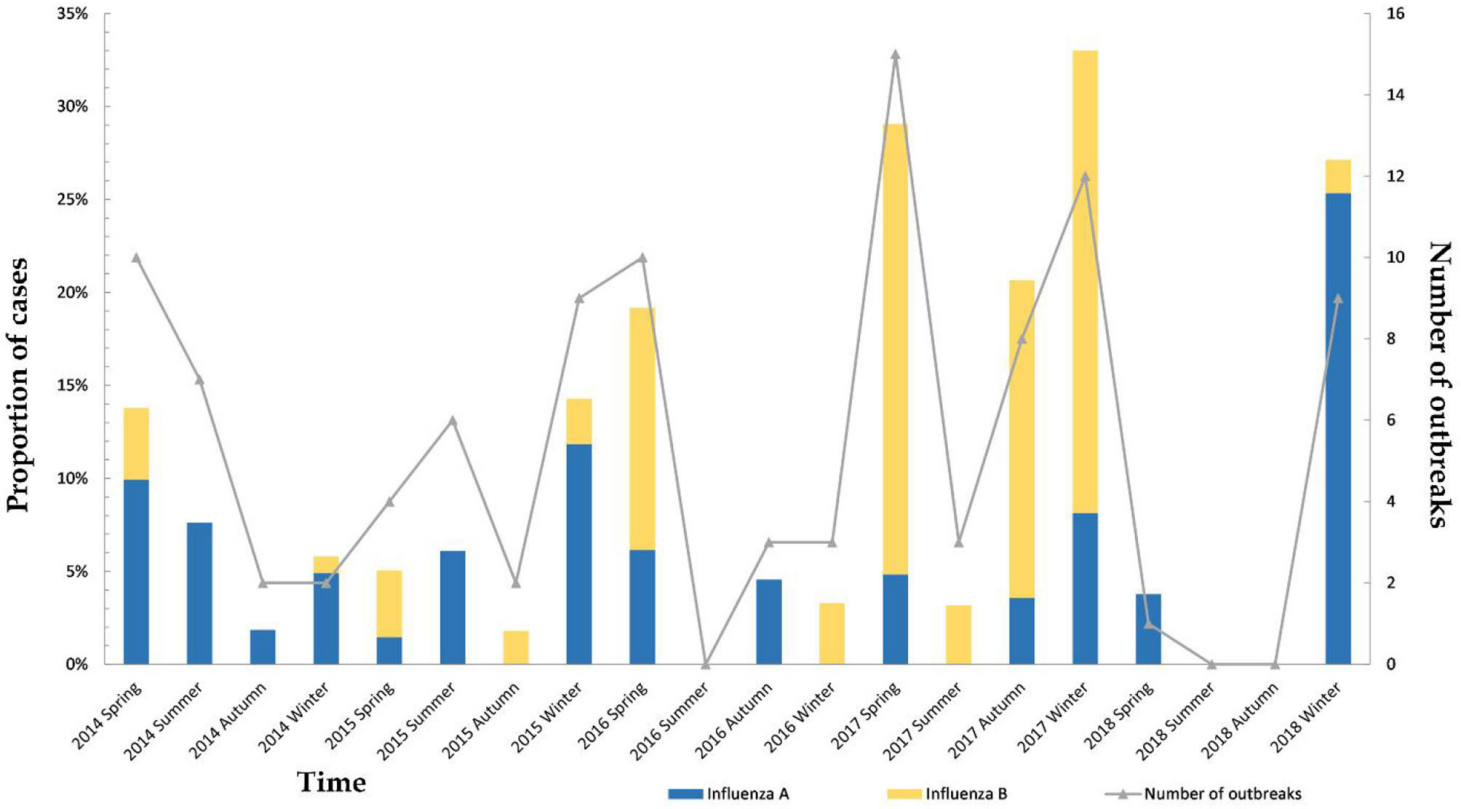
Seasonal distribution of influenza A and B (number of cases and outbreaks).

Figure 3 shows the seasonal distribution of influenza A and B from the spring of 2014 to the winter of 2018. Epidemic peaks of influenza cases and cluster outbreaks were seen in the spring or winter, with the highest number of cluster outbreaks being in the spring of 2017 and the highest proportion of influenza infection being in the winter of 2017. Figure 3 shows that 2017 experienced the worst influenza outbreak in the past 5 years.

### Spatial Distribution of Influenza

**Figure 4:**
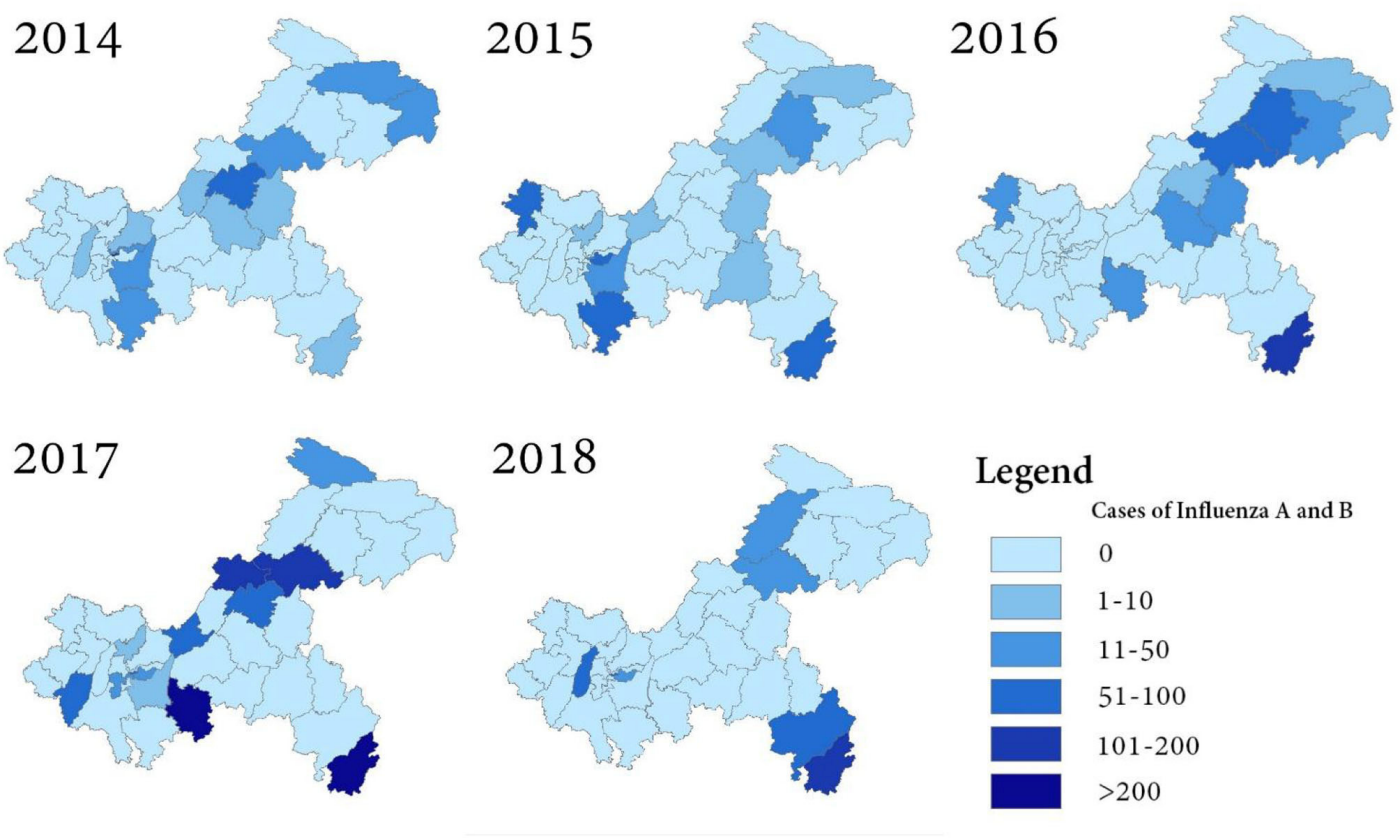
Spatial distribution of influenza A and B.

As shown in Figure 4, influenza A and B affected the 38 districts in various degrees in Chongqing, China. The results showed clear annual spatial differences among these districts. Xiushan district, which is in the southeast corner of Chongqing, and Wanzhou district, located in the northeastern part of Chongqing, were continuously affected by influenza from 2014 to 2018. Wanzhou district has the largest economy away from the major city proper. It also has one of the highest population densities apart from the major city proper. Yuzhong district, which is in the center of Chongqing and with one of the highest populations, was hit hard by influenza during the 5 years. Yuzhong district, being the smallest district with the fastest growing economy in Southwest Chongqing, was also hit hard by influenza during the 5 years.

The cases of influenza A and B infection were more abundant in Northeast Chongqing, with a random pattern of district distribution. Higher concentrations of cases occurred in Northcentral Chongqing in 2014, 2016, and 2017. The number of cases in Southwest Chongqing significantly exceeded that of the surrounding areas, especially in 2014, 2015, and 2017. An increased number of influenza infection cases could be observed in Southeast Chongqing throughout the surveillance period.

### Spatial Autocorrelation Analysis

**Figure 5:**
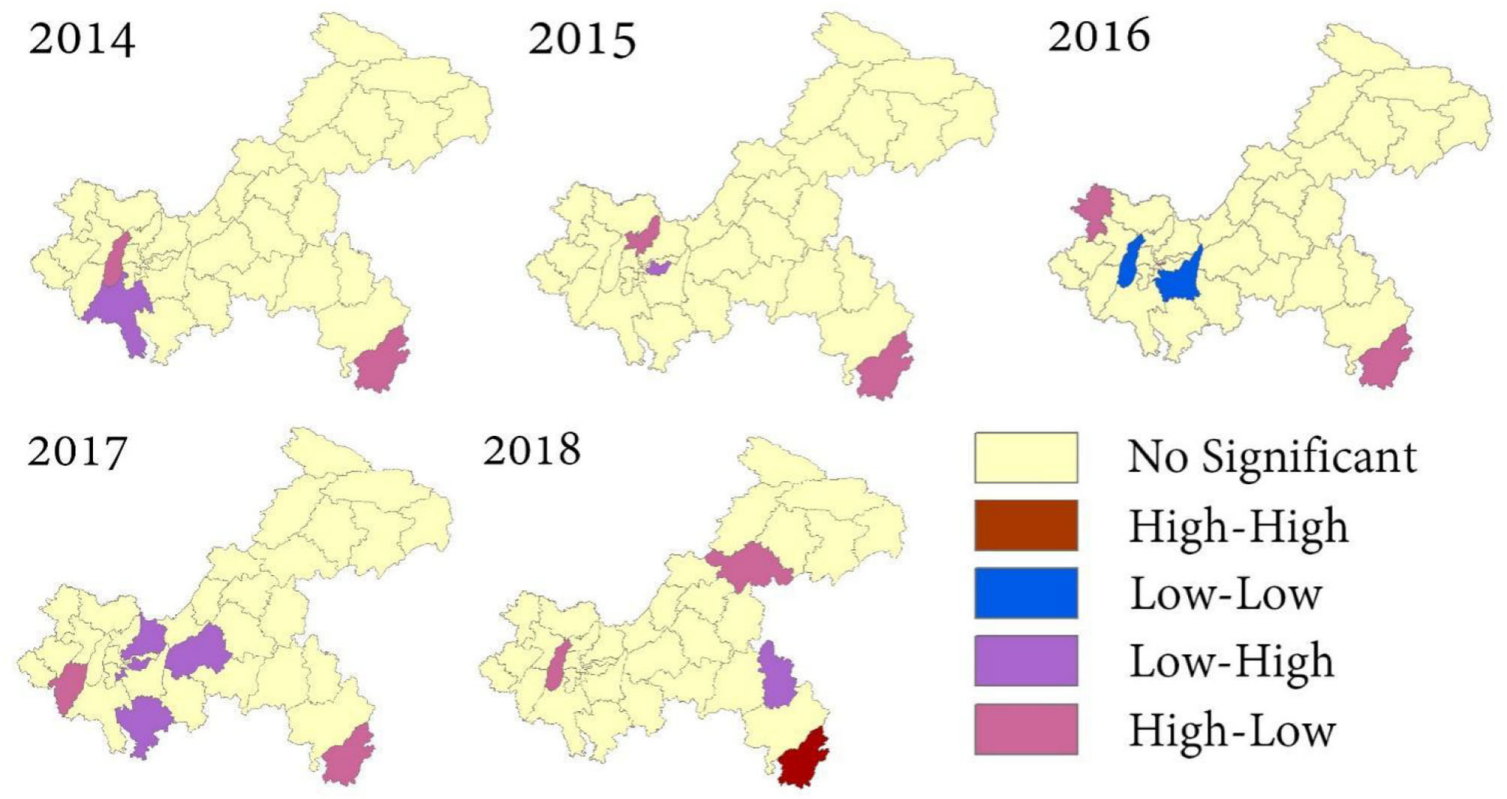
Local spatial autocorrelation of influenza in Chongqing.

Except for 2018 (Moran's I = 0.3702, P = 0.004), the global spatial autocorrelation of influenza incidence in the 5 years was not significant. The results are shown in Table 3. According to the annual LISA maps of influenza A and B incidence, the local autocorrelation detected one high-high, two low-low, eight low-high, and 11 low-high clusters (Figure 5). A notable detail that the high-high and high- low clusters were most frequently observed in Xiushan district throughout the 5 years.

**Table 3:** Global spatial autocorrelation of influenza in Chongqing.

**Year**	**Moran's, I Index**	**Moran's Z-score**	**Moran's I *P*-value**	**Mean**	**SD**
2014	−0.0850	−0.6420	0.261	−0.0263	0.0915
2015	−0.0573	−0.4083	0.405	−0.0264	0.0758
2016	−0.0352	−0.0875	0.465	−0.0266	0.0974
2017	0.0275	0.8245	0.177	−0.0252	0.0639
2018	0.3702	4.9662	0.004	−0.0290	0.0804

## Discussion

Influenza is one of the most prevalent diseases that poses a significant risk of severe or fatal outcomes, especially in preschool and school-aged children (17, 33–38). Increased knowledge about the distribution characteristics of school cluster outbreaks and clinical features could aid in predicting the peak time, outbreak area, student groups, and subtypes or lineages. Thus, effective interventions, such as a school-based vaccination program, could be implemented (9, 39, 40). The present study aimed to explore the epidemiological and clinical features of influenza in preschool and school-aged children in Chongqing, China.

From 2014 to 2018, influenza A and influenza B alternately dominated the number of cluster outbreaks in schools in Chongqing. In 2017, when influenza B dominated, the number of cluster outbreaks reached its peak and declined in 2018. Similar to previous findings, influenza A viruses usually stay as the dominant viruses (41–43), while influenza B viruses could emerge as prevailing viruses from time to time, especially among the very young population (44, 45). However, B/Victoria was the dominant strain throughout 2017. The widespread outbreak of 2017 spring could be explained by the annual seasonal epidemic of B/Victoria. In addition, the unusually high prevalence for 2017 autumn and winter could partly be explained by the extremely cold and dry weather that year and the low flu vaccination coverage in Chongqing. After the severe outbreak in 2017, the number of cluster outbreaks significantly declined in the next year, as particularly seen in influenza B. The drop, consistent with Dahlgren's findings (46), could be partly explained by the quickly enhanced immunity for type B viruses among children (47).

In this study, the general characteristics were compared, and the results showed that students in special needs school were at risk compared with kindergarten students. B/Victoria viruses and unidentified B lineage were found to be risk factors compared with H1N1, winter was a protective factor compared with spring, and living in rural areas was a risk factor of influenza infection.

In line with prior results, younger children were more likely to become infected (33, 48, 49), probably because the immune function of these children were not mature enough. Herein, students in special-needs schools require more attention than kindergarten students, and such finding is rarely found in previous literature. One hypothesis is that students in special-needs school are bound to make more contact with the faculty. The service staff in similar institutions were proven to be strong carriers of influenza viruses in Louise's and Arielle's studies (50, 51).

B/Victoria virus also had a higher infection rate among preschool and school-aged students (52). In particular, the only death detected in the 5 years was related to B/Victoria virus. This result further supported the previous idea of influenza B/Victoria was more infective among younger children (53, 54).

However, several studies reported that the difference in influenza infection between rural and urban areas was not significant or even more cases in urban areas than in rural areas (55, 56). The unexpected finding is that children in rural areas had a higher incidence of influenza infection and living in rural areas was a risk factor of influenza. This observation highlighted the need for influenza intervention and prevention measures in rural schools in the Chongqing area. One possible explanation is that urban schools are at a higher level of epidemic prevention and control, with few clusters of outbreaks. The measures could be vaccination, home quarantine, and wearing masks. Given that rural children's parents work in urban areas, many rural schools are boarding schools, which not only add to the contact of students but also reduce the home quarantine rate. In addition, rural students are less likely to be vaccinated than urban students.

In the analysis of clinical features of influenza A and B, the difference between the two groups were compared. Previous evidence supported that children with influenza B had greater adjusted odds of presenting with headache, sore throat, and abdominal pain. Cough, nasal discharge, and dizziness were also identified to be significantly more common in patients with influenza B (12, 57). The reason why symptoms appeared more frequent in influenza B cases was the ability of older children to localize symptoms. Older children were more likely to report headache, sore throat, and myalgia (57). Given that no firm evidence could support the hypothesis, more data are required to determine the possible reasons for this phenomenon in the future. In combination of the fact that the only fatal case in 5-year surveillance was caused by influenza B virus, seasonal influenza B viruses possibly did not receive enough attention in Chongqing, especially among preschool and school-aged children.

The seasonal distribution of influenza A and B showed a specific pattern of peaking in spring and winter according to the previous findings in Chongqing (58). Though Li Qi's study reported another peak in June and July, any extremely high incidence of severe influenza in the summer was not found. One possible explanation is that preschool and school-aged children spend most of their summer holidays at home, thus effectively reducing their contact with each other. However, due to the shortness of winter break in Chinese schools and the high prevalence of influenza in the winter, the incidence of school-based cluster outbreaks in winter remains a high level. School closure has been applied in Hongkong as an intervention to control influenza epidemics (39, 59). It is one of the effective measures to alleviate seasonal and pandemic influenza. With the support of previous data, this study recommends that Chongqing Education Bureau considers gradual closure and closure of all schools within the same county when excess absenteeism occurs [62].

The spatial distribution of influenza cases maps and spatial autocorrelation analysis confirmed the necessity of influenza intervention in Xiushan district. The complex geography, poor healthcare, and location in the southernmost part of Chongqing are responsible for the high incidence of epidemics in Xiushan. Moreover, Xiushan district, which is located in Southeast Chongqing, is an important channel connecting Chongqing to the southeast coastal economic circle. As a traffic stronghold, the population movement and increased contact made it easy to spread and invade Xiushan from Southeast China. Besides, schools in Xiushan are mostly boarding schools, and the closed environment may create conditions for the spread of influenza outbreaks. Thus far, the epidemic in Xiushan has not received sufficient attention, and no relevant studies have focused on the epidemic situation of influenza in this region. This study could enable the local government to pay increased attention to the influenza outbreaks among preschoolers and school-aged children in Xiushan. More proactive interventions, such as organizing vaccinations at the school level and school closures, should be taken in Xiushan.

## Limitations and Strengths

This study innovatively monitored influenza epidemic data in Chongqing City by spatiotemporal analysis and further explored the epidemic and spatial distribution characteristics of influenza, thus providing theoretical basis for the prevention and control of influenza in Chongqing. However, some areas of the study could still be improved, such as more data to provide a clearer trend analysis of the pattern of epidemic influenza. Besides, symptom data were collected within 3 days of diagnosis, and further data should be included given the effect of clinical treatments, such as drugs, and poor accuracy of reporting symptoms by young children. The data in this study were collected on cluster outbreak level, and the original data we collected were not case by case. Future research can consider collecting case by case data to allow for the application of more robust statistical methods. In addition, subsequent spatiotemporal analysis of preschool and school-aged children may consider including cases of sporadic influenza to improve the accuracy of the study. Finally, the reported findings are limited to Chongqing. Future studies should consider a national survey.

## Conclusions

In this study, the sociodemographic and clinical characteristics and temporal and spatial distribution of influenza A and B were explored, contributing to the differential diagnosis and school-based intervention of seasonal influenza A and B viruses. The results provide more information for health workers to control seasonal influenza epidemics among preschool and school-aged children in Chongqing.

## Data Availability Statement

The raw data supporting the conclusions of this article will be made available by the authors, without undue reservation.

## Author Contributions

MY and XM: conceptualization. HZhu and XM: methodology and software. RO, MY, QZ, and HL: validation, visualization, and supervision. XM: formal analysis, writing–original draft preparation and writing–review and editing. HZha: investigation, resources, and data curation. MY: project administration and funding acquisition. All authors have read and agreed to the published version of the manuscript.

## Conflict of Interest

The authors declare that the research was conducted in the absence of any commercial or financial relationships that could be construed as a potential conflict of interest.

## Publisher's Note

All claims expressed in this article are solely those of the authors and do not necessarily represent those of their affiliated organizations, or those of the publisher, the editors and the reviewers. Any product that may be evaluated in this article, or claim that may be made by its manufacturer, is not guaranteed or endorsed by the publisher.
